# Reviewer Acknowledgment

**DOI:** 10.1002/rmb2.70007

**Published:** 2025-12-26

The publication of invaluable papers in the Reproductive Medicine and Biology depends on the prompt careful review of submitted manuscripts. We would like to thank the Editorial Board members the Editorial Advisory Board members and the following experts for reviewing manuscripts from November 1, 2024, to October 31, 2025.

Aiping Mao

Akari Kusamoto

Akemi Koshiba

Akihito Horie

Akihito Morita

Akiko Sankoda

Akimasa Takahashi

Ayako Muraoka

Ayano Yamaya

Ayumu Ito

Chisato Kunitomi

Christine Rousset‐Jablonski

Dilip Kumar Swain

Eiji Nishio

Eri Maeda

Esmat Mangoli

Fuminori Kimura

Fuminori Taniguchi

Gaku Shimoi

Haruhiko Kanasaki

Hidetaka Tasaki

Hideyuki Kobayashi

Hirohisa Kyogoku

Hiroki Fujimoto

Hiroki Noguchi

Hiroki Takeuchi

Hiromitsu Shirasawa

Hiroshi Harayama

Hiroshi Ishikawa

Hiroshi Kishi

Hiroshi Koike

Hiroyuki Tomari

Hitomi Nakamura

Ilse Goovaerts

Isao Tamura

Isao Tsuji

Iwaho Kikuchi

Jota Maki

Katsuya Sato

Kazuhiro Kawamura

Kazuhisa Tomita

Kei Miyamoto

Keisuke Okada

Kenichi Kashimada

Kenichiro Hiraoka

Kenichiro Moromura

Kenji Ezoe

Kenji Imai

Kenshiro Hara

Kentaro Takezawa

Kentaro Tanemura

Kiyotaka Kawai

Kohei Ogawa

Koichi Kyono

Koji Hayakawa

Koji Sugiura

Kosuke Taniguchi

Kotaro Kitaya

Kouhei Sugimoto

Kouji Komatsu

Kuniaki Ota

Maki Kusumi

Manabu Satoh

Mari Nomiyama

Martha Valdivia

Masahiko Nakatsui

Masahiro Ashizawa

Masahito Tachibana

Masanori Ono

Masashi Iijima

Masashi Takamura

Masato Kobanawa

Masato Yoshihara

Masatoshi Ooga

Mayuko Kato

Meryem Pekcan

Michio Kitajima

Mitsunori Matsuo

Mitsuru Komeya

Mitsutoshi Yamada

Miyuki Harada

Morita Akihito

Nadia Sheibak

Natsuki Miyake

Nobuhiko Suganuma

Norichika Ueda

Noritaka Fukunaga

Noritoshi Enatsu

Nozomi Takahashi

Osamu Yoshino

Qi‐En Yang

Rie Fukuhara

Riffat Bibi

Ryota Kobayashi

Satoru Takamizawa

Satoshi Ando

Satoshi Kishigami

Satoshi Mizuno

Satoshi Tsukamoto

Sayaka Wakayama

Seido Takae

Seiji Sumigama

Seiko Matsuo

Seung‐Yup Ku

Shinichiro Fukuhara

Shinnosuke Komiya

Shinnosuke Kuroda

Shirasawa Hiromitsu

Shoichiro Iwatsuki

Shuai Zhang

Shun Sato

Shunichiro Tsuji

Shuntaro Ikeda

Suguru Sato

Sung‐Ki Lee

Susumu Tanaka

Taisuke Mori

Takahide Hayano

Takashi Horikawa

Takashi Umehara

Takayuki Yamochi

Takehiro Hiraoka

Takeshi Kajihara

Takeshi Sato

Tatsuma Yao

Tatsuro Furui

Tatsuya Kobayashi

Teppei Takeshima

Tetsuaki Hara

Tetsuya Mizutani

Tomohiro Ishii

Tomoko Ichikawa

Tomoko Nakamura

Tomomi Kotani

Tomomoto Ishikawa

Toshifumi Takahashi

Toshio Hamatani

Toshiya Matsuzaki

Toshiyuki Iwahata

Tsuyoshi Baba

Tsuyoshi Takiuchi

Udayanga Kankanam Gamage

Yasuhiko Kamada

Yasuhisa Yamashita

Yasushi Hirota

Yasushi Kawano

Yasushi Takai

Yasushi Yumura

Yasuyuki Mio

Yasuyuki Negishi

Yodo Sugishita

Yoko Nagayasu

Yonglun Fu

Yoshihara Kosuke

Yoshiharu Nakaoka

Yoshikazu Kitahara

Yoshimasa Asada

Yoshimitsu Kuwabara

Yoshimitsu Wada

Yoshinori Takeda

Yosuke Ono

Yosuke Tarumi

Yuji Hirao

Yukihiro Umemoto

Yumiko Miyazaki

Yuri Yamamoto

Yusuke Sako

